# Dual-Wavelength Terahertz Metasurfaces with Independent Phase and Amplitude Control at Each Wavelength

**DOI:** 10.1038/srep34020

**Published:** 2016-09-23

**Authors:** Jun Ding, Ningning Xu, Han Ren, Yuankun Lin, Weili Zhang, Hualiang Zhang

**Affiliations:** 1 ECE Department, University of Massachusetts, Lowell, MA 01854, USA; 2 School of ECE, Oklahoma State University, Stillwater, OK 74078, USA; 3 EE Department, University of North Texas, Denton, TX 76207, USA; 4 Physics Department, University of North Texas, Denton, TX 76203, USA

## Abstract

We have designed, fabricated and characterized dual-wavelength metasurfaces that function at two assigned terahertz wavelengths with independent phase and amplitude control at each wavelength. Specifically, we have designed a dual-wavelength achromatic metasurface-based deflector deflecting the incident wave to the same direction at two selected wavelengths, which has circumvented the critical limitation of strong wavelength dependence in the planar metasurface-based devices caused by the resonant nature of the plasmonic structures. As a proof of concept demonstration, the designed dual-wavelength achromatic deflector has been fabricated, and characterized experimentally. The numerical simulations, theoretical predictions, and experimental results agree very well with each other, demonstrating the property of independently manipulating the phase profiles at two wavelengths. Furthermore, another unique feature of the designed metasurface is that it can independently tailor both the phase and amplitude profiles at two wavelengths. This property has been numerically validated by engineering a metasurface-based device to simultaneously generate two diffraction orders at two desired wavelengths.

Metamaterials have attracted significant attention in the past decade due to a number of fascinating phenomena not existing in nature, such as negative refraction^1^,^2^,^3^, invisible cloaking^4^,^5^, super-resolution imaging^6^. Recently, the concept of metasurface^7^,^8^,^9^, a novel type of planar metamaterials with subwavelength thickness, has been demonstrated to achieve important functionalities as in conventional optical systems by introducing abrupt phase shifts at the thin interfaces with metallic or dielectric resonators^10^,^11^. Compared to the conventional bulky optical devices that rely on gradual phase accumulation through a long propagation distance to tailor the wavefront, the unique approach of utilizing metasurfaces to achieve the phase discontinuities promises devices with flatness and small footprint beyond the scope of conventional components. Up until now, V-shape and C-shape metallic resonators and their complementary or Barbinet-inverted counterparts have been widely applied in metasurfaces^10^,^12^,^13^,^14^. In order to achieve the full 2π phase discontinuity coverage, the cross-polarized component of the incident light is used.

With the ability of the full 2π phase shift control by carefully engineering each unit cell in a supercell, a number of emerging applications of metasurfaces, such as wave plates^15^,^16^,^17^,^18^, beam steering^10^,^19^,^20^, lensing^21^,^22^,^23^,^24^,^25^, hologram^26^,^27^,^28^,^29^, active metasurfaces with phase-change materials^30^,^31^, etc., have been demonstrated through numerical simulations and experiments. Significant effort has been devoted to realize metasurfaces with characteristics such as broadband and independent polarization control^13^,^22^,^32^,^33^,^34^. However, very few investigations have been conducted to design metasurface structures that could function in a multi-wavelength manner (e.g., deflecting the wavefront to the same direction or focusing to the same focal plane at two or more wavelengths), circumventing the strong wavelength dependence limitation of the planar devices, which is one of the most critical limitations. Very recently, one-dimensional (1-D) achromatic metasurfaces based on coupled rectangular dielectric resonators (RDRs) in *x*-axis were theoretically and experimentally demonstrated^35^,^36^, which could work at three different telecommunication wavelengths overcoming the chromatic aberrations. In this design, a unit cell consisted of two RDRs was utilized, and then aperiodic arrays with desired phase profiles were formed to achieve 1-D achromatic deflector and flat lens at three wavelengths. The desired phase and amplitude profiles for all unit cells at the three designed wavelengths were optimized through extensive simulations by adjusting the width of each RDR and the coupling distance between the two RDRs, thus the devices function in one direction only and the desired phase and amplitude profiles for each wavelength are not independently or simultaneously controlled. Besides the 1-D nature of the devices, due to the limitation of the structure size and coupling between the two RDRs in such a unit cell, the ratio between two different working wavelengths could be limited (e.g., <1.5 in refs 35 and 36). Except these few demonstrations of achromatic metasurfaces, most of the metasurfaces demonstrated so far are designed to work at a single wavelength with the capabilities to tailor the phase profile or phase-amplitude profiles or phase-polarization profiles simultaneously of the transmitted/reflected EM waves in a single layer or few-layer metasurface structures^37^,^38^,^39^,^40^,^41^.

In this paper, we design and fabricate a novel metasurface structure that can independently manipulate both the phase and amplitude at two terahertz wavelengths with a flexible wavelength ratio λ_2_/λ_1_ (the ratio could vary from around 1.25 to as large as 3 or even larger), which could be advantageous in multispectral terahertz imaging and sensing^42^,^43^. The building block of the designed structure is composed of a two-layer structure separated by a polyimide spacer and backed by a thick silicon substrate. The top layer is composed of metallic C-shape split-ring resonators (CSRR) and the bottom ground layer consists of Complementary CSRRs (CCSRR) with a circular hole in the middle of the unit cell, which is referred as Modified CCSRR or MCCSRR in the following sections. The required phase and amplitude profiles for the dual-wavelength devices usually cannot be simultaneously satisfied at both wavelengths in the terahertz region by simply putting together two regular resonators (CSRRs or CCSRRs) due to the strong interference and coupling between them. This critical issue is addressed by the introduced circular hole in the MCCSRR, which could maintain the properties of both CSRR and CCSRR. In such a construction, both the phase and amplitude behaviors of the designed metasurfaces can be simultaneously manipulated at the two selected wavelengths independently. As a proof of concept example, we first demonstrate the design, fabrication, and measurement of a dual-wavelength achromatic terahertz deflector that can deflect the normal incident wave to the same direction at two assigned wavelengths. The experimentally measured results agree well with the numerical simulations and theoretical predictions. The designed metasurface also exhibits a relatively broadband property by deflecting the normal incident wave to the anomalous directions guided by the generalized Snell’s Law at a wide vicinity of the two selected wavelengths. To the best of our knowledge, this is the first time a dual-wavelength terahertz metasurface-based device with these features has been designed and experimentally validated. Furthermore, to demonstrate the unique feature of manipulating both the phase and amplitude simultaneously at two wavelengths, we also construct a metasurface device that can generate two diffraction orders at two working wavelengths. The performance of the resulting device has been verified numerically. The numerical results agree well with the theoretical prediction. The designed scheme could open up new avenues for realizing dual-wavelength metasurfaces and their applications in practical systems.

## Results

### The building block for the dual-wavelength devices

**Figure 1:**
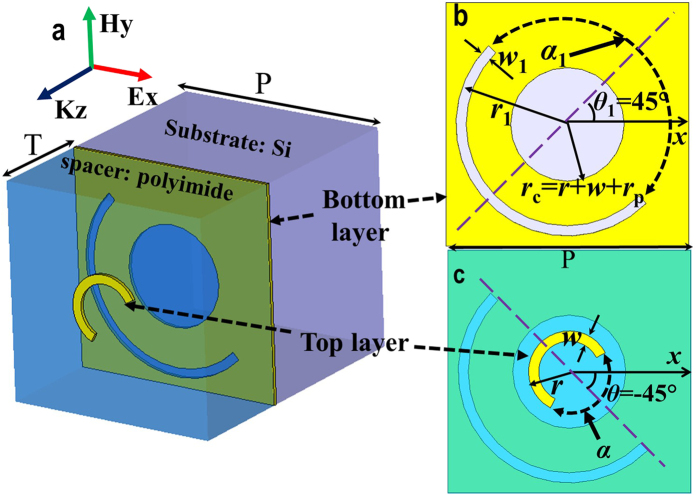
(**a**) Schematic of a building block in the designed dual-wavelength achromatic metasurface. It is composed of a top layer (CSRR) and a bottom ground layer (MCCSRR) separated by a polyimide spacer; (**b**,**c**) represent the CCSRR with a circular hole in the middle (MCCSRR) and CSRR, respectively. The opening angle, width, orientation with respect to *x*-axis, and outer radius of CSRR (CCSRR) are *α* (*α*_1_), *w* (*w*_1_), *θ* (*θ*_1_), and *r* (*r*_1_), respectively. The radius for the circular hole in (**b**) is *r*_c_ = *r *+ *w *+ *r*_p_. In the simulations, *θ* and *θ*_1_ are set as either 45° or −45°.

To realize the dual-wavelength terahertz devices, the CSRR and MCCSRR are utilized as the key elements in a building block or unit cell because they individually feature several desired properties in addition to the full 2π phase discontinuity coverage, including broadband^13^, simultaneous control of phase and amplitude^44^, strong magnetic moment^45^. Due to the strong interference and coupling, the required phase and amplitude profiles for the dual-wavelength devices cannot be both satisfied at both working wavelengths by just simply putting together any two resonators (e.g., CSRR and CCSRR, referring to Supplementary Figures S1–S4 in the Supplementary Information (SI)), which indicates that the performance of one resonator could be degraded by the other resonator. Fortunately, this critical issue is addressed by the designed building block consisted of a top layer CSRR and a bottom layer MCCSRR, the schematic of which is illustrated in Fig. 1a. The circular hole introduced on the bottom ground layer plays a critical role: it could restore the performance of CSRR located at the top layer and keep the desired response of the CCSRR on the bottom ground layer almost intact (see Supplementary Figure S5). As a result, the behavior of each resonator can be independently controlled with a proper size of the circular hole. The two layers are separated by a polyimide spacer of thickness T = 20 μm with a permittivity of 2.96 and a loss tangent of 0.091. Here the spacer thickness T is chosen for the ease of fabrication. Resonators at both top layer and bottom ground layer are made from 200 nm thick aluminum with the conductivity of 3.72 × 10^7^ S/m.

Figure 1b,c show the top views of the MCCSRR and CSRR, respectively. The whole structure is backed by a bulky layer of Silicon with a permittivity of 11.9. As studied thoroughly in refs 10, 13 and 44, the 2π phase coverage could be realized by tuning the opening angle *α*/*α*_1_ and the outer rim radius *r*/*r*_1_ of the SRR/CSRR (with orientation *θ*/*θ*_1_ either 45° or −45° with respect to the *x*-axis). More interestingly, the transmission amplitude could also be adjusted by rotating the orientation *θ*/*θ*_1_. In this designed metasurface, we set the periodicity P to be 120 μm, the outer rim radius *r* (*r*_1_) to be 20 μm (55 μm), the width *w* (*w*_1_) for CSRR (MCCSRR) to be 5 μm, and the *r*_p_ of the circular hole to be 3 μm. The orientation *θ*/*θ*_1_ is set as either 45° or −45°. The required phase profiles are obtained by varying the open angle *α* (*α*_1_) and *θ* (*θ*_1_). These variables can provide many more degrees of freedom to achieve much more complex phase and amplitude profiles.

### Design theory for the dual-wavelength achromatic devices

**Figure 2:**
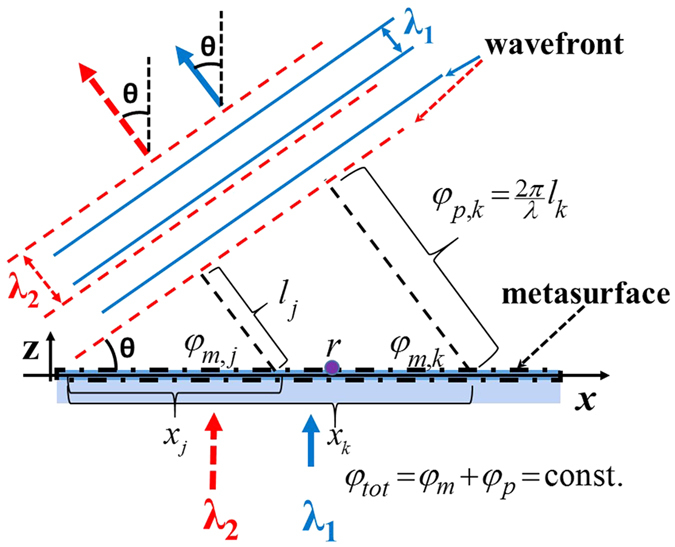
Schematic of the wave propagation in a dual-wavelength achromatic metasurface. It is required that the total phase accumulation *φ*_*tot*_ should be kept constant at the two wavelengths.

In order to design an achromatic metasurface-based device, the phase accumulation in the propagation path after passing through the metasurface needs to be considered in addition to the abrupt phase shift imparted at the metasurface. As shown in Fig. 2, to ensure the achromatic functionality, the total accumulated phase 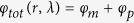 should be kept constant at the selected wavelengths^35^. *φ*_*m*_ is the abrupt phase discontinuity imparted at position *r* by the metasurface; *φ*_*p*_ is the phase accumulated in the wave propagation path after passing through the metasurface. The abrupt phase change *φ*_*m*_ is usually introduced by a nano-resonator element, which is the only variable considered in previous periodic phase-gradient metasurfaces^10^,^13^, and is independent of the position *r*. *φ*_*p*_ depends on the position *r* and the wavelength *λ* and can be calculated as 
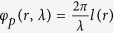
, where *l*(*r*) is the propagation distance from the metasurface at position *r* to the propagation wavefront.

Therefore, to keep 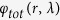 unchanged, the goal of devising an achromatic metasurface is to tailor the dispersion of *φ*_*m*_ to compensate for *φ*_*p*_ according to the following equation


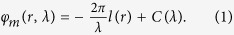


In equation (1), 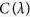 can take on any values and can be treated as an additional degree of freedom in the selection of the resonator elements. The key of this approach is to construct a resonator array (i.e., metasurface) with the desired phase profile for *φ*_*m*_ in equation (1), which depends on both position *r* and wavelength *λ* to compensate the propagation phase *φ*_*p*_. It is noted that the constructed array should be generally aperiodic^35^, but it could be periodic with special combinations of *l*(*r*) and *λ*.

### Design of a dual-wavelength achromatic deflector

As a proof of concept demonstration, we designed a 1-D metasurface-based achromatic deflector in the XZ plane, then, the whole structure in the XZ plane is repeated along the Y direction. It is worth mentioning that the unit cell shown in Fig. 1a can be positioned in both the *x*- and *y*-directions independently, thus 2-D metasurfaces can be realized using the designed metasurface unit cell. The spectral responses of the unit cell are numerically simulated by using CST Microwave Studio. An *x*-polarized plane wave is illuminated from the bottom side propagating in the *z*-direction, as shown in Fig. 1a, while the *y* component of the transmitted electric field is detected. The basic unit of the dual-wavelength achromatic metasurface is designed to adjust the required cross-polarized transmission phase shift 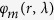 at two selected wavelengths to satisfy equation (1) with a fixed 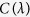. The achromatic deflector is designed to deflect the normally incident light to the angle 
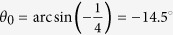
 at two selected terahertz wavelengths (λ_1_ = 240 μm and λ_2_ = 750 μm). The desired spatially varying phase functions are defined by:


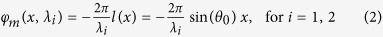


where *x* is the spatial coordinate of the metasurface, starting from the center of the first unit cell, i.e., 0.5P.

**Figure 3:**
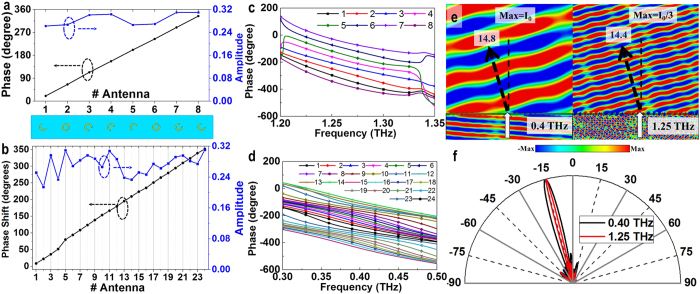
(**a,b**) Phase shift and the transmission amplitude for the CSRR at 1.25 THz and MCCSRR at 0.4 THz, respectively; (**c**,**d**) the phase profiles for the supercells in (**a**,**b**), respectively; (**e**) simulated *y*-component electric field distributions in the XZ plane for the *x*-polarized normal incidence at 0.4 and 1.25 THz; (**f**) normalized far-field responses at 0.4 and 1.25 THz.

For the top layer, substituting λ_1_ = 240 μm and P = 120 μm into equation (2), 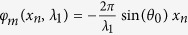, where *x*_*n*_ = 0.5P+ (*n* − 1) P is the center of *n*^th^ unit cell, we achieve the required abrupt phase shift between two adjacent cells as 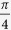. In this special case, the top layer metasurface happens to be a periodic structure consisting of 8-resonator supercells like a phase gradient metasurface^10^. For the bottom ground layer, substituting λ_2_ = 750 μm and P = 120 μm into equation (2), we obtain the required abrupt phase shift for each cell as 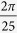, which happens to be a period structure with 25-resonator supercells. In order to demonstrate the dual-wavelength deflector as a general case and reduce the computation burden, we choose a periodic structure with 24-building-block supercells for both layers, which means a periodic structure for λ_1_ and a truncated periodic structure for λ_2_ (could be treated as an aperiodic structure in ref. 35). Specifically, for the top layer, we chose four resonators with nearly identical transmission amplitude and a 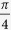 phase increment for deflecting the incident wave to −14.5° at λ_1_ = 240 μm or *f*_1_ = 1.25 THz. The geometrical parameters of the resonators are *α* = 147°, 27°, 71°, and 107° with corresponding orientation *θ* = 45°, −45°, −45°, and −45°, respectively, shown in the lower part of Fig. 3a. Following that, another four resonators with an additional π phase shift are achieved by flipping these four resonators over the *x*-axis or changing the sign of the orientation angle of these resonators, which agrees with the observation in refs 10 and 13. After placing all 8 resonators together along the *x*-axis at the surface with sorted phase shift, a linear gradient phase profile can be obtained as shown in Fig. 3a. According to the numerical calculation, it is noticed that these 8 resonators also exhibit similar equal phase interval profiles from 1.2 to 1.35 THz as illustrated in Fig. 3c, leading to a relatively broad bandwidth.

After the design of the top layer, we chose 24 resonators located at the bottom layer with similar transmission amplitude and a 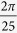 phase increment to form a supercell for deflecting the incident wave to −14.5° at λ_2_ = 750 μm or *f*_2_ = 0.4 THz. The detailed geometrical parameters of these cells are listed in Supplementary Table S1. Fig. 3b shows the transmission amplitude and phase shift for these 24 resonators with sorted phase shift. It can be observed in Fig. 3b that the phase shift distribution among these resonators is almost linear except that there is a small discrepancy between cell numbers 4 and 5, corresponding to the last and first cells of the 24-building-block supercell, which is due to the truncation of the periodicity. Although with this small phase shift discrepancy, from the simulation and measurement, we observe that the results are in good agreement with the design goals. This is in consistence with the statement in ref. 13 that the exact phase increments between two adjacent cells are not strictly necessary as long as keeping the trend of the phase shift. Figure 3d plots the phase profile of the 24-resonator supercell in Fig. 3b from 0.3 to 0.5 THz, which also exhibits a relatively broadband property.

To demonstrate the performance of the designed dual-wavelength deflector, we first numerically simulate the structure consisting of 24 building blocks with a total size of 2880 μm × 120 μm (see Methods). Figure 3e illustrates the simulated *y*-component electric field distributions with the *x*-polarized normal incident plane wave propagating along *z*-axis at 0.4 and 1.25 THz. It can be seen that the normal incident wave at 0.4 and 1.25 THz is deflected to −14.8° and −14.4°, respectively, which matches very well with the design goal of −14.5°. The dual-wavelength deflecting functionality is further confirmed by the normalized far-field responses illustrated in Fig. 3f. The *y*-component electric field distributions at the vicinity of the two selected wavelength are plotted in Supplementary Figure S6. It can be seen that the uniform wavefronts are well deflected following the generalized Snell’s Law.

### Experimental verification of the dual-wavelength achromatic deflector

**Figure 4:**
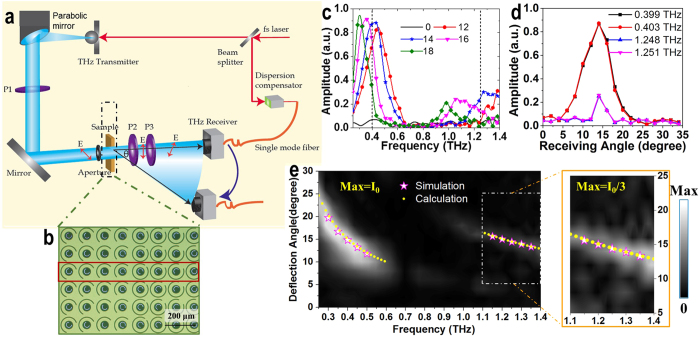
Schematic diagram of (**a**) experimental setup and (**b**) image of a portion of the fabricated sample. (**c**) Measured transmission amplitudes of the deflector from 0.25 to 1.4 THz under *x*-polarized normal incidence as the receiving angles are 0°, 12°, 14°, 16° and 18°. (**d**) Measured transmission amplitudes of the deflector against the receiving angle at 0.3988, 0.4025, 1.2477, and 1.2513 THz. (**e**) Experimental results of the deflection effect with respect to the frequency (*x*-axis) and deflection angle (*y*-axis). The simulated and calculated results are plotted as pink pentagrams and yellow dots, respectively. The inset shows the portion from 1.1 to 1.4 THz with enlarged intensity.

To experimentally verify the performance of the designed dual-wavelength metasurface-based deflector, the aforementioned metasurface device composed of 24-building-block supercells was fabricated (see Methods) and measured. The supercell is repeated 5 times in the *x*-direction, and the whole structure along the *x*-axis is repeated 120 times in *y*-direction, thus the whole size of the fabricated sample is 1.44 cm × 1.44 cm. As the schematic diagram of the experimental arrangement shown in Fig. 4a, a fiber based angular resolved terahertz time-domain spectroscopy (THz-TDS) was employed to measure the light deflection of the dual-band metasurface sample^46^. The terahertz radiation was generated by a photoconductive switch-based antenna, optically pumped by an 800 nm femtosecond laser. Then the terahertz beam was collected by a parabolic mirror and passed through a 90° polarizer. A linearly polarized terahertz wave is reflected and filtered by an aperture with 1.6 cm opening before illuminating the sample. Another two polarizers with 0° and 45° installed after the sample enable a cross-polarization transmission measurement. The signal is referenced by a bare silicon wafer measured with co-polarization. The linear electric fields at each step are labeled as a red arrow shown in the figure. Finally, the signal was detected by a fiber based terahertz receiver, optically pumped by the same femtosecond laser as the transmitter. By mounting the receiver on a metal holder, it allows an accurate arc rotation on its center. With the sample fixed in the center, the measurement was carried out angle-resolved by every 2° from 0° to 34°. The image of a portion of the fabricated sample is shown in Fig. 4b. The 8-cell supercell for top layer (as highlighted in Fig. 4b) is the same as in Fig. 3a. The deflection effect from 0.25 to 1.4 THz can be characterized in a single THz-TDS measurement at a specific detection angle. Figure 4c shows the measured transmission amplitude against frequency at the receiving angles of 0°, 12°, 14°, 16° and 18° (the complete measured data sets are plotted in Supplementary Figure S7), and Fig. 4d shows the measured transmission amplitude against receiving angle extracted from Supplementary Figure S7 at 0.399, 0.403, 1.248, and 1.251 THz.

### Metasurface-based THz device generating two diffraction orders at two wavelengths

**Figure 5:**
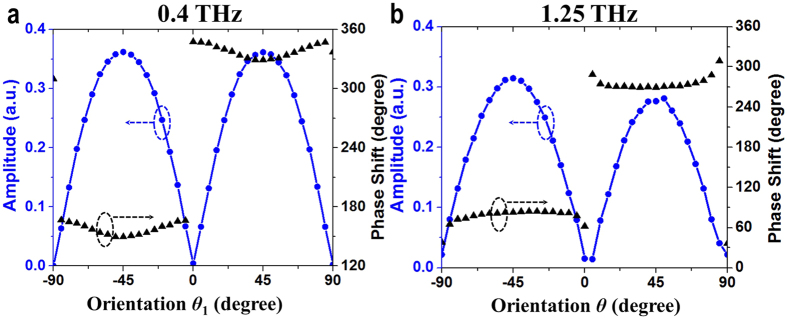
Angular dependence of the phase and amplitude of the unit cell of the CSRR-MCCSRR structure with (*α* = *α*_1_ = 71°) by (**a**) rotating the *θ*_1_ at 0.4 THz, (**b**) rotating *θ* at 1.25 THz. (*r* = 20, *r*_1_ = 55, and *w* = *w*_1_ = 5 unit: μm).

Another unique feature of the designed metasurface structure is that it is capable of simultaneously controlling both phase and amplitude of the transmitted wave at two desired wavelengths. Figure 5 illustrates the simulated amplitude and phase variations of a MCCSRR (CSRR) at different orientation angles *θ*_1_ (*θ*) from −90° to 90° at 0.4 THz (1.25 THz) with *α*_1_ = 71° (*α* = 71°) (this is a representative unit cell of the 24-resonator supercell in the above achromatic deflector). As can be seen from Fig. 5a (Fig. 5b), the amplitude varies generally as |sin(2*θ*_1_)| (|sin(2*θ*)|) while the phase remains almost constant in two separated rages of [−90°, 0°] and [0°, 90°], with an abrupt phase shift of π at *θ*_1_ (*θ*) = 0, which is very similar as in the single CSRR structure reported in ref. 44. The little phase discrepancy by varying the orientation *θ*_1_ in Fig. 5a and the little amplitude variation by varying *θ* in Fig. 5b can be remedied by adjusting the radius *r*_c_, thus, the amplitude profiles can be manipulated independently at two wavelengths. Combined with the analysis in section I of SI, it can be concluded that both phase and amplitude responses can be independently controlled at the two selected wavelengths by utilizing the designed metasurface structure.

**Figure 6:**
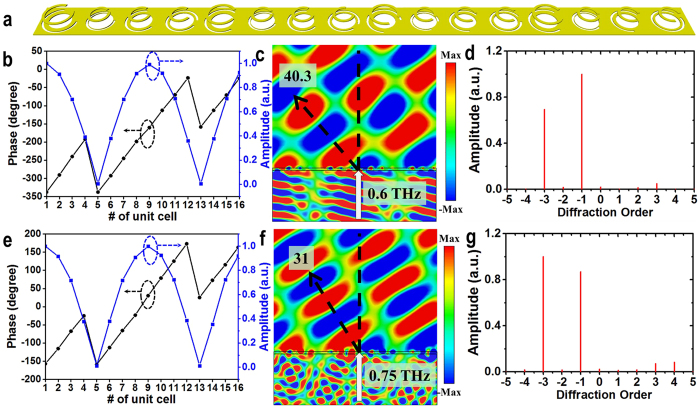
(**a**) Schematic diagram of the designed device to generate two diffractions at two wavelengths. (**b**,**e**) Required phase and amplitude profiles at 0.6 and 0.75 THz, respectively. (**c**,**f**) Simulated *y*-polarized electric field distributions at 0.6 and 0.75 THz, respectively. (**d**,**g**) Retrieved diffraction orders through Fourier Transformation at 0.6 and 0.75 THz, respectively.

In order to validate this attractive feature discussed above, we numerically study another terahertz device based on the designed metasurface structure. It is engineered to generate two diffraction orders (−1^st^ and −3^rd^) at two desired wavelengths with a much smaller wavelength ratio of 1.25 (λ_1_ = 400 μm, λ_2_ = 500 μm). Notice that this smaller wavelength ratio is chosen to demonstrate that the designed dual-wavelength metasurface can support a large range of wavelength ratios (a large wavelength ratio >3 has been demonstrated in the previous example). To achieve this function, the transmission through the metasurface should be in general expressed as equation (3)^44^,


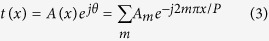


where P is the periodicity of the unit cell, *x* is the spatial coordinate, and *m* is an integer denoting the diffraction order. For achieving two diffraction orders at two wavelengths, it is required that both the phase and amplitude of the transmission in each unit cell need to be carefully chosen at both working wavelengths (which have not been demonstrated before). As mentioned in previous sections, the required phase shifts can be obtained by varying the opening angle *α*/*α*_1_, the outer rim radius *r*/*r*_1_ of CSRR/MCCSRR, and the circular radius *r*_c_, while the required amplitudes can be achieved by rotating the orientation *θ*/*θ*_1_. Figure. 6a illustrates the schematic diagram of the 16-building-block supercell of the designed device, which contains four different building blocks with evenly spanned phase shifts from 0 to π at a step of 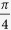 at both working wavelengths. The required phase and amplitude for each unit cell are achieved by rotating the orientations of the four basic unit cells. Specifically, the geometrical parameters of the four CSRRs are *α* = 55.56°, 26.54°, 69.58°, and 88.14° with corresponding outer rim radius *r* = 38 μm, 27.64 μm, 28.16 μm, and 27.7 μm, respectively; while the geometrical parameters of the four MCCSRRs are *α*_1_ = 98°, 60.4°, 115°, and 30° with corresponding circular radius *r*_c_ = 27.9 μm, 33 μm, 27 μm, and 29 μm, respectively, and *r*_1_ = 44 μm, 36 μm, 40 μm, 40 μm, respectively (note that the orientations for CSRR and MCCSRR are all 45° in the four basic unit cells except *θ*_1_ = −45° in the fourth basic unit cell). The periodicity P, the width *w* for the CSRR and the width *w*_1_ for the MCCSRR are set to be 100 μm, 7 μm and 5 μm, respectively. The detailed parameters of *α*_1_ and *θ*_1_ for MCCSRR are listed in Supplementary Table S2. Based on the designed metasurface structures, the resulting phase and amplitude profiles satisfying equation (3) at 0.6 and 0.75 THz are plotted in Fig. 6b,e, respectively. It is worth emphasizing that the circular hole in the MCCSRR plays a critical role for finely tuning the required phase and amplitude profiles. In the simulation, an *x*-polarized plane wave is normally illuminated from the substrate side onto the designed metasurface propagating in the *z*-direction. Figure 6c,f show the *y*-polarized electric field distributions at 0.6 and 0.75 THz, respectively. Interfered wavefronts (due to the interference between the two diffraction orders) are observed at the two working frequency wavelengths. And the interfered wavefronts are deflected to −40.3° and −31° at 0.6 and 0.75 THz, respectively, agreeing very well with the theoretical calculations of −38.7° and −30°, respectively, by the generalized Snell’s Law^10^. Furthermore, the information of the diffraction orders at the two working frequency bands can be retrieved through applying Fourier Transformation to the extracted field distributions (along *x*-axis at a distance of 1000 μm away from the metasurface). The retrieved results are shown in Fig. 6d,g, where two dominant diffraction orders (*m* = −1 and −3) at the two wavelengths can be clearly observed, verifying the performance of the designed device.

## Discussions

Dual-wavelength metasurfaces have been demonstrated to independently manipulate both phase and amplitude profiles at two selected terahertz wavelengths (the ratio between the two working wavelengths can be varied from around 1.25 to 3 or even larger). As the first example, a dual-wavelength achromatic deflector aiming to circumvent the strong wavelength dependence has been designed and experimentally demonstrated, which can deflect the normal incident wave to one anomalous direction at λ_1_ = 240 μm and λ_2_ = 750 μm. Besides, the designed deflector has exhibited a relatively broadband property by deflecting the normal incident wave to the directions in coincidence with the generalized Snell’s Law at a wide vicinity of the two working wavelengths. The experimental results agreed very well with the simulated and calculated results. In addition, another metasurface-based device that can generate two diffraction orders has been numerically validated at two wavelengths (λ_1_ = 400 μm, λ_2_ = 500 μm), which has demonstrated the promising property of the designed metasurface to independently control both the phase and amplitude at two desired wavelengths. Moreover, since the required phase and amplitude profiles at the two working wavelengths can be independently manipulated in both *x*- and *y*- directions, our approach can be generally utilized to engineer 2D dual-wavelength devices, such as 2D lenses and axicons. Overall, the designed scheme can provide new tools to control the propagation of terahertz waves and pave the way towards developing more sophisticated dual-wavelength devices with other fascinating functionalities in the future.

## Methods

### Simulations of the dual-wavelength achromatic deflector

The achromatic deflector is numerically simulated by using CST Microwave Studio. In the simulations, the periodic boundary conditions are applied in both the *x*- and *y*-directions, replicating the fabricated structure in which the 24-building-block supercell is repeated 5 and 120 times in the *x*- and *y*-directions, respectively (since the whole structure is required to be 1arger than 1 cm × 1 cm in the measurement). Open boundary conditions are applied in the *z*-direction with vacuum of a certain thickness being the spacer on both the metasurface and substrate sides.

### Fabrication of the dual-wavelength achromatic deflector

The metasurface sample was made of aluminum on the silicon substrate with a polyimide spacer. First, the 200 nm thick bottom ground layer was patterned with MCCSRR on the 640 μm silicon substrate by conventional photolithography technique and thermal evaporation. After a 20 μm thick polyimide spacer is uniformly spin-coated on the bottom ground layer, the top 200 nm metallic CSRR patterns are aligned in the center with the bottom MCCSRR.

## Additional Information

**How to cite this article**: Ding, J. *et al*. Dual-Wavelength Terahertz Metasurfaces with Independent Phase and Amplitude Control at Each Wavelength. *Sci. Rep.* **6**, 34020; doi: 10.1038/srep34020 (2016).

## Supplementary Material

Supplementary Information
