# Supplementary material for: Dual-Wavelength Terahertz Metasurfaces with Independent Phase and Amplitude Control at Each Wavelength
Source: Sci Rep. 2016 Sep 23;6:34020. doi: 10.1038/srep34020 (PMC5034287; doi:10.1038/srep34020)
Supplement: Supplementary Information [file srep34020-s1.pdf]

Supplementary Information for

Dual-Wavelength Terahertz Metasurfaces with Independent Phase and Amplitude Control at Each Wavelength

Jun Ding[1,‡], *Ningning Xu*[2,‡], *Han Ren*[3], *Yuankun Lin*[3,4], *Weili Zhang*[2], and *Hualiang Zhang*[1,*]

[1]ECE Department, University of Massachusetts Lowell (One University Ave., Lowell, MA 01854, USA)

[2]School of ECE, Oklahoma State University (Stillwater, OK 74078, USA)

[3]EE Department, University of North Texas (3940 N. Elm St., Denton, TX 76207, USA)

[4]Physics Department, University of North Texas (1155 Union Circle, #311427, Denton, TX 76203, USA)

* Corresponding Author: HL Zhang. Email: hualiang_zhang@uml.edu.

‡ These authors contributed equally to this work.

Section I. The critical role of the circular hole in the MCCSRR

In the following discussion, the spacer and the substrate are the same as in Figure 2, typical values of P = 120 μm, α = 107 °, θ = ±45^0, r = 20 μm, α_1 = 97 °, θ_1 = ±45^0, r_1 = 55 μm, and $w = w_1$ = 5 μm are chosen to demonstrate the different performances of different structures. We will explore unit cells with different combinations of CSRRs and CCSRRs, e.g., (1) structures with only CSRR or only CCSRR cases, (2) dual-CSRRs structure in a single layer, (3) dual-CCSRRs structure in two layers, and (4) CSRR-MCCSRR structure in two layers with/without the circular hole, and it can be concluded that the circular hole in the CSRR-MCCSRR structure plays a critical role in the design of the dual-wavelength metasurfaces.

1. Structures with only CSRR and only CCSRR cases

Without the loss of generality (and because the magnitude response is more critical), we plot the magnitude responses in Figure S1 ("Both" means to keep both top and bottom layers, "NoTop" means only the bottom layer is presented, and "NoBot" means only the top layer is presented). Figure S1a shows that top layer resonator has very little effect to the whole structure in the lower frequency band (at around 0.4 THz): the magnitude responses for the "Both" and the "NoTop" cases are almost identical, while the magnitude response for the "NoBot" case is almost 0. Figure S1b plots the magnitude responses in the higher frequency band (at around 1.25 THz). It can be seen that the magnitude response for the "Both" case conforms to the trend of that for the "NoBot" case, indicated by the two vertical dash lines. Furthermore, the magnitude response for the "NoTop" case is much smaller than those for the "Both" and the "NoBot" cases at 1.25 THz. Thus, we may

conclude that the magnitude responses at 0.4 and 1.25 THz are mainly determined by the bottom and top resonators, respectively.

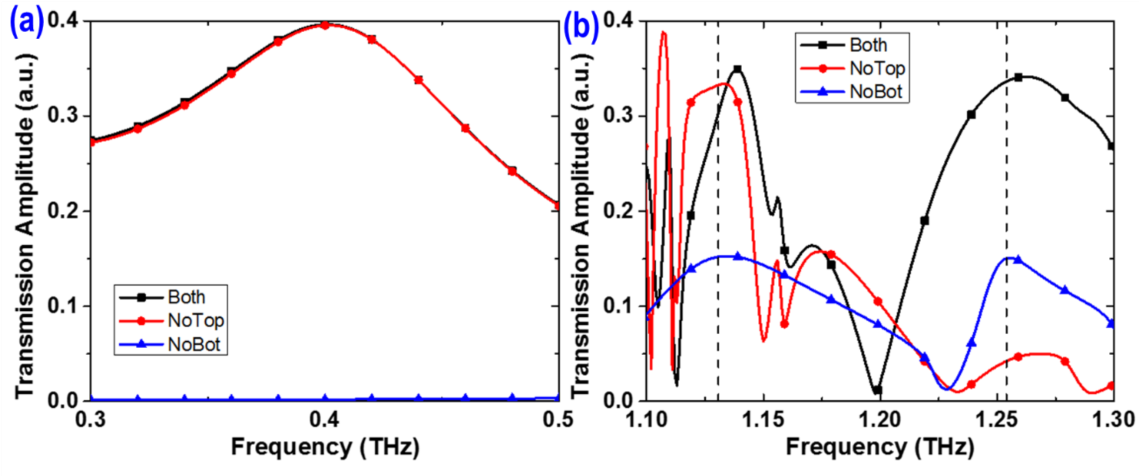

Figure S1: Magnitude responses for "Both", "NoTop", and "NoBot" cases in the (a) lower frequency band (around 0.4 THz) and (b) higher frequency band (around 1.25 THz).

The *x*-polarized electric field distributions for different cases are plotted in Figure S2 with *y*-polarized incident wave from substrate side. Figures S2a and S2b are almost identical, and Figures S2c and S2d have slight difference, which indicate that the resonances for the "Both" case come from corresponding resonances of the "NoTop" and "NoBot" cases.

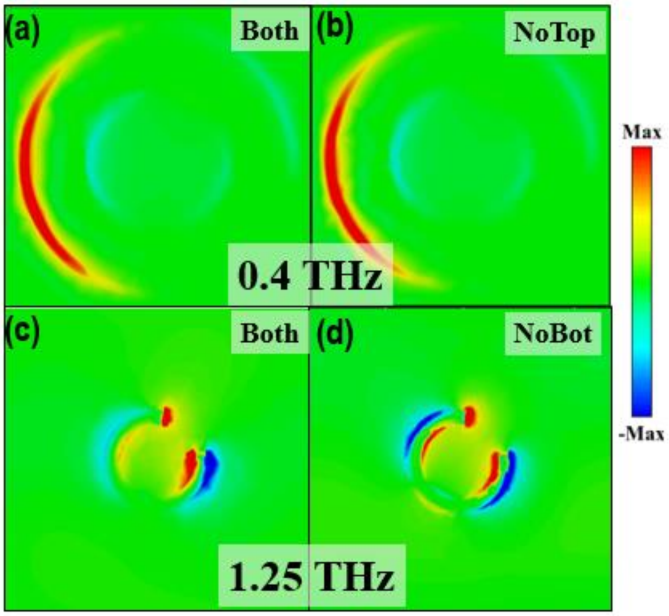

Figure S2: *x*-polarized electric field distributions for (a) "Both" case at 0.4 THz, (b) "NoTop" case at 0.4 THz, (c) "Both" case at 1.25 THz, and (d) "NoBot" case at 1.25 THz with *y*-polarized incident wave from the substrate side.

2. Dual-CSRRs structure in one layer

In this dual-CSRRs structure, two CSRRs are located in the same layer: one outer larger CSRR and one inner smaller CSRR as shown in the inset of Figure S3d. From Figure S3a and S3c, we can observe that the desired properties (such as broadband, an additional π phase shift and nearly identical cross-polarized transmittance after flipping over the *x*-axis) of CSRR are well kept in the lower frequency band ([0.35, 0.7] THz) by varying the outer radius of CSRR. However, Figure S3b and S3d show that the cross-polarized transmittance has large variation in the higher frequency band ([1.2, 1.3] THz), and the additional π phase shift and nearly identical cross-polarized transmittance cannot be achieved after flipping over the *x*-axis, thus the 2π phase coverage and almost identical transmission magnitude

cannot be satisfied in the higher frequency band with this dual-CSRRs structure. Similar conclusions can be obtained for the dual-CCSRRs structure in one layer.

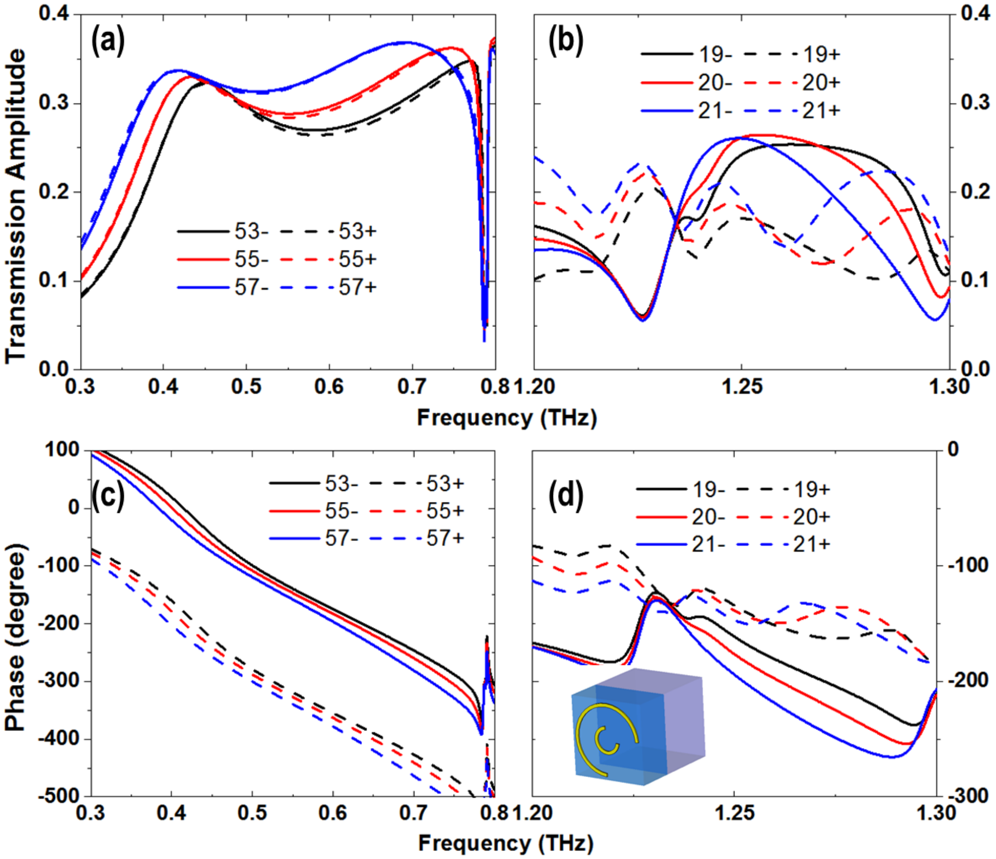

Figure S3. (a) and (c) Cross-polarized transmission (t_{xy}) amplitude and phase at the lower frequency band ([0.3, 0.8] THz) with different radiuses of the outer CSRR, respectively, for the dual CSRRs structure; (b) and (d) Cross-polarized transmission (t_{xy}) amplitude and phase at the higher frequency band ([1.2, 1.3] THz), respectively. In the legends, the number means the outer radius of the CSRR, and the "-" and "+" represent the orientations of the SRR as -/+ 45°, respectively. The schematic of the dual-CSRRs structure is shown in the inset of (d).

3. Dual-CCSRRs structure in two layers

In this dual-CCSRRs structure, two CCSRRs are located in two layers separated by a spacer: a top metallic layer perforated with a smaller CSRR and a bottom metallic layer perforated

with a larger CSRR, as shown in the inset of Figure S4c. From Figure S4a and S4b, we can observe that cross-polarized transmissions are extremely small (almost negligible) under this scenario. However, Figure S4c and S4d show that the co-polarized reflections are near unit, and the phases are almost unchanged (not shown here). According to the analysis in[1], the 2π phase coverage cannot be obtained in either frequency band with this dual-CCSRRs structure.

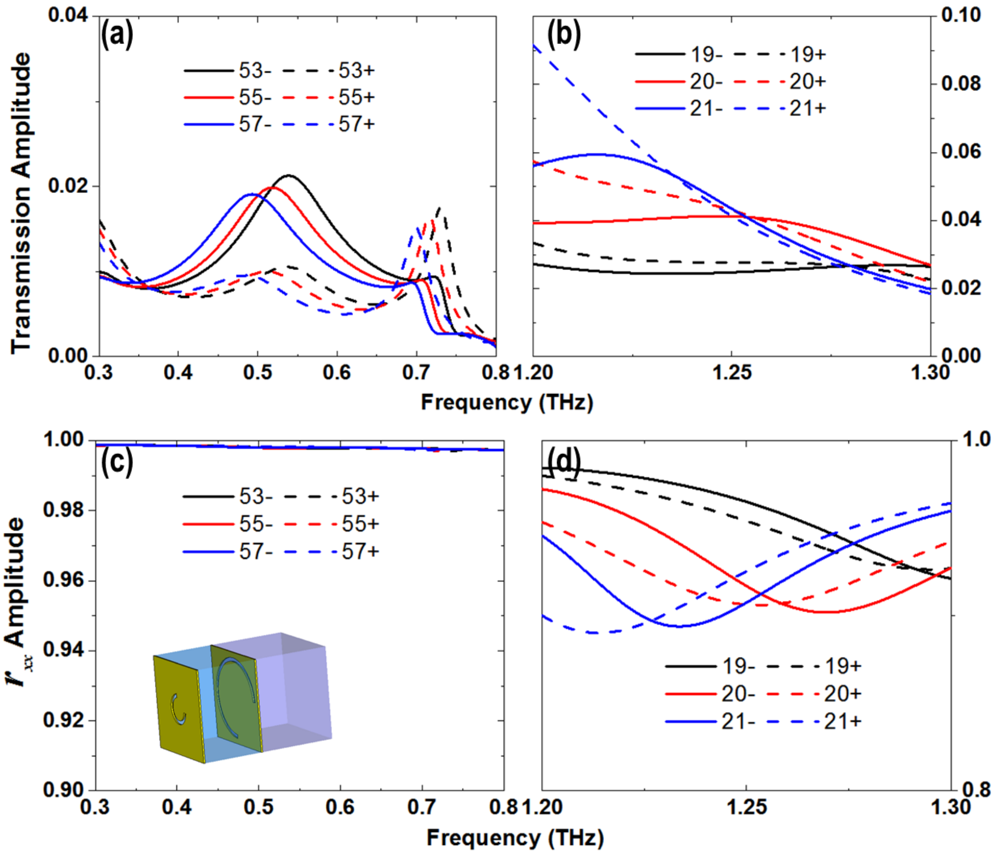

Figure S4. (a) and (b) Cross-polarized transmission (t_{xy}) amplitude for the dual-CCSRRs structure; (c) and (d) Co-polarized reflection (r_{xx}) amplitude. In the legends, the number means the radius of the SRR and the "-" and "+" represent the orientations of the CCSRR as -/+ 45°, respectively. The schematic of a dual-CSRR is shown in the inset of (c).

4. The CSRR-MCCSRR structure in two layers

The CSRR-MCCSRR structure (i.e., the proposed structure) is comprised of a top layer of CSRR and a bottom layer of MCCSRR corresponding to the higher and lower frequency bands, respectively, as shown in the inset of Figure S5d. The outer radiuses for the top layer CSRR and bottom MCCSRR are r and r_1, respectively. Figure S5a and S5c show the cross-polarized transmission (t_{xy}) amplitude and phase at the lower frequency band ([0.3, 0.7] THz) with various radiuses of the circular hole ($r_c = r + w + r_p$), respectively. When the radius of the circular hole is 0 ("NoHole" case) or small (e.g., $r_c = 5$ μm), the magnitude and phase responses are almost indistinguishable, overlapping with the "NoTop" case, which indicates the top layer has little effect in the lower frequency band. When the r_c increases, the transmission amplitude decreases slowly around the resonance (i.e., 0.45 THz in this case, close to the design frequency) and decreases rapidly away from the resonance (e.g., > 0.5 THz), and the phase keeps almost unchanged with the radius comparable to the $r = 20$ μm (e.g., $r_c \leq 30$ μm). If r_c is larger compared to the $r = 20$ μm and $r_1 = 55$ μm (e.g., $r_c = 40$ μm), both transmission amplitude and phase exhibit large discrepancies even around the resonance.

Figure S5b and S5d show the cross-polarized transmission (t_{xy}) amplitude and phase at the higher frequency band ([1.2, 1.3] THz) with various radiuses of the circular hole, respectively. When the radius of the circular hole is 0 ("NoHole" case) or small (e.g., $r_c \leq$ 20 μm), the magnitude is very small, thus the phase is irrelevant. When the r_c is increased to 30 μm, the transmission amplitude is enhanced at the designed frequency 1.25 THz from almost 0 to around 0.3; when r_c keeps increasing to 40 μm, the magnitude decreases. As can be seen from Figure S5d that the phase keeps a small discrepancy for a proper size of

the circular hole, such as 28 μm and 30 μm. Through extensive simulations, we observe that the 2π phase coverage can be achieved by varying α ($\theta = \pm 45°$) with high transmission amplitude of around 0.3 with a proper size of the circular hole, which is almost independent of the bottom MCCSRR. Furthermore, the desired properties of CSRR in the CSRR-MCCSRR structure can be maintained as in a single CSRR structure.

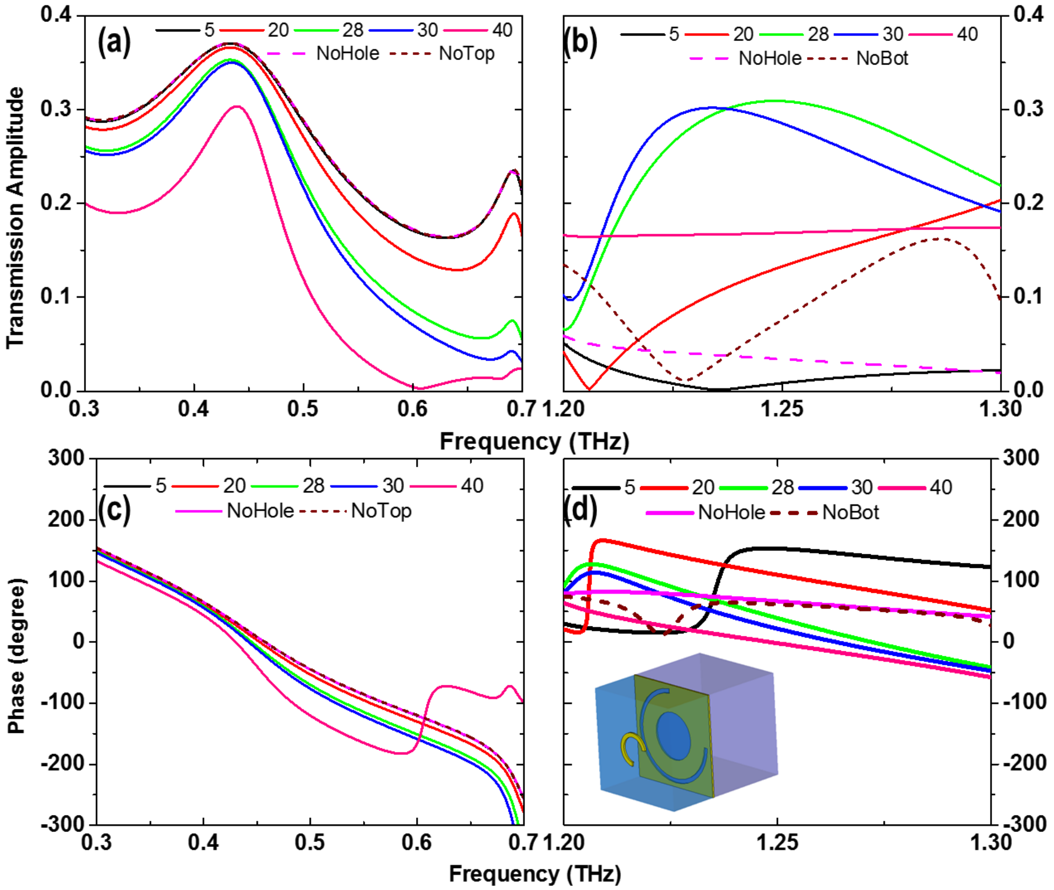

Figure S5. (a) and (c) Cross-polarized transmission (t_{xy}) amplitude and phase at the lower frequency band ([0.3, 0.7] THz), respectively, for the CSRR-MCCSRR structure; (b) and (d) Cross-polarized transmission (t_{xy}) amplitude and phase at the higher frequency band ([1.2, 1.3] THz), respectively. In the legends, the number means the radius of the circular hole, "NoHole" means there is no hole in the bottom layer, and "NoTop" ("NoBot") indicates that there is no top (bottom) layer in the structure. The schematic of a CSRR-MCCSRR is shown in the inset of (d).

From the above analysis, it can be concluded that the required phase profiles can be achieved at two design wavelengths (frequencies) independently with a proper size of the circular hole in a CSRR-MCCSRR structure, while these requirements cannot be satisfied in other combinations, therefore, the circular hole plays a critical role in the design of the proposed dual-wavelength metasurfaces and related devices.

Section II. The relatively broadband property of the proposed dual-wavelength metasurface-based deflector

As can be seen from Figure 3c and 3d, it is expected that the deflector could work in a wide frequency (wavelength) range in the vicinity of the two working frequencies (wavelengths). The simulated E_y fields at several frequencies around the two working frequencies (i.e., 0.3, 0.4 0.5, 1.2, 1.25, and 1.3 THz) are plotted in Figure S6 with an *x*-polarized incident wave. The incident waves are well deflected following the generalized Snell's Law[1], and uniform transmitted wavefronts are observed.

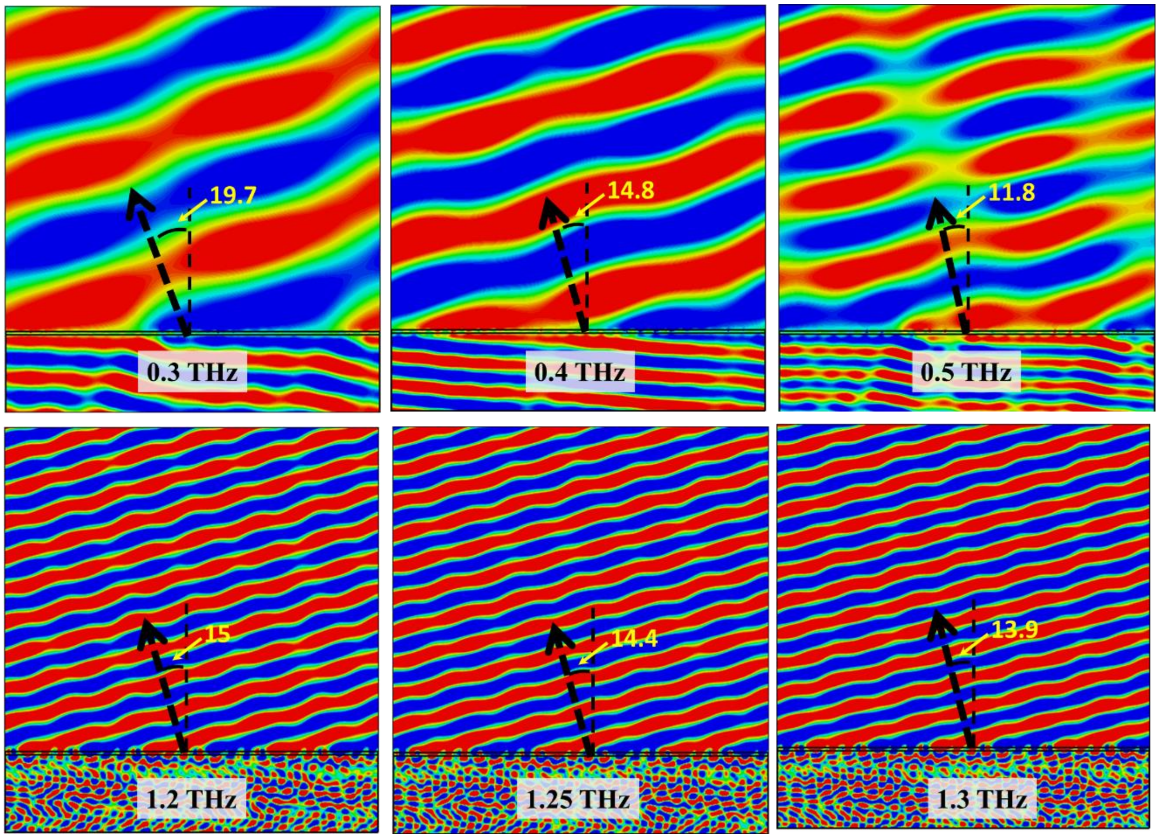

Figure S6. Simulated *y*-component electric field distributions in the XZ plane for the *x*-polarized normal incidence at 0.3, 0.4, 0.5, 1.2, 1.25, and 1.3 THz.

Table S1 Opening angle (α_1) and orientation (θ_1) for the 24-resonator supercell (r_1 = 55 μm, r = 20 μm, w = w_1 = 5 μm, r_p = 3 μm)

Cell	#1	#2	#3	#4	#5	#6	#7	#8	#9	#10	#11	#12
α_1(°)	113	131	153	177	25	40	59	67	79	87	97	105
θ_1(°)	45	45	45	45	-45	-45	-45	-45	-45	-45	-45	-45
**Cell**	**#13**	**#14**	**#15**	**#16**	**#17**	**#18**	**#19**	**#20**	**#21**	**#22**	**#23**	**#24**
α_1(°)	123	143	167	180	17	30	47	61	71	81	90	100
θ_1(°)	-45	-45	-45	-45	45	45	45	45	45	45	45	45

Section III. Complete measured data sets

Figure S7 plots the measured transmission amplitude of the proposed dual-wavelength deflector from 0.25 to 1.4 THz under the x-polarized normal incidence as the receiver scans from 0 ° to 34 ° with a step of 2 °.

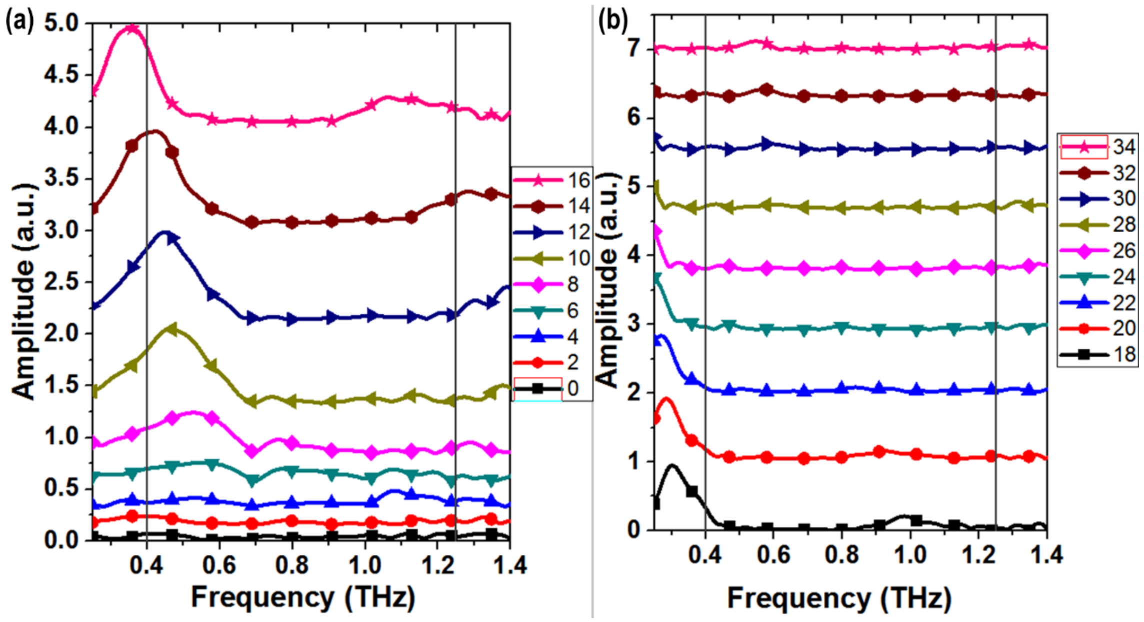

Figure S7. The measured transmission amplitude of the fabricated dual-wavelength deflector.

Section IV. Parameters for the device generating two diffraction orders at two wavelengths

Table S2 Opening angle (*α_1*) and orientation (*θ_1*) for the 16-resonator supercell

Cell	#1	#2	#3	#4	#5	#6	#7	#8
α_1(°)	98	60.4	115	30	98	60.4	115	30
θ_1(°)	45	33.75	22.5	-11.25	0	11.25	22.5	-33.75
**Cell**	**#9**	**#10**	**#11**	**#12**	**#13**	**#14**	**#15**	**#16**
α_1(°)	98	60.4	115	30	98	60.4	115	30
θ_1(°)	-45	-33.75	-22.5	11.25	0	-11.25	-22.5	33.75

1 Yu N, Genevet P, Kats MA, Aieta F, Tetienne J-P, Capasso F *et al.* Light Propagation with Phase Discontinuities: Generalized Laws of Reflection and Refraction. *Science* 2011; **334**: 333–337.
